# The Prevalence and Context of Alcohol Use, Problem Drinking and Alcohol-Related Harm among Youth Living in the Slums of Kampala, Uganda

**DOI:** 10.3390/ijerph17072451

**Published:** 2020-04-03

**Authors:** Monica H. Swahn, Rachel Culbreth, Laura F. Salazar, Nazarius Mbona Tumwesigye, David H. Jernigan, Rogers Kasirye, Isidore S. Obot

**Affiliations:** 1 School of Public Health, Georgia State University, P.O. Box 3984, Atlanta, GA 30302, USA; lsalazar1@gsu.edu; 2 School of Public Health, Makerere University, Kampala P.O. Box 7062, Uganda; naz@musph.ac.ug; 3 Johns Hopkins Bloomberg School of Public Health, Baltimore, Maryland, MD 21205, USA; djernigan@jhu.edu; 4 Uganda Youth Development Link, Sir Apollo Kaggwa Rd, Kampala P.O. Box 12659, Uganda; kasiryer@yahoo.com; 5 Department of Psychology, University of Uyo, Uyo 520003, Nigeria; obotis@gmail.com

**Keywords:** alcohol use, adolescent health, global health, binge drinking, unsafe sex, violence

## Abstract

Background. The purpose of this paper is to investigate the prevalence and context of alcohol use, problem drinking and alcohol-related harm among boys and girls in the slums of Kampala, Uganda. Methods. The Kampala Youth Survey is a cross-sectional study conducted in 2014 among youth (ages 12–18 years) living in the slums of Kampala (*n* = 1133) who were participating in Uganda Youth Development Link (UYDEL) centers. Chi-square tests were used to determine differences in alcohol use behaviors between 1) gender (boys vs. girls), and 2) alcohol use behaviors between problem drinkers and non-problem drinkers, stratified by gender. Results. Among all participants (*n* = 1133), the prevalence of any alcohol use in the past 12 months was 31% (*n* = 346). A higher percentage of girl drinkers reported having sex in the past month, without a condom (57.9%) due to alcohol consumption, compared to boy drinkers (41.9%) ($\;\chi^{2}$ = 8.09, *df* = 1, *p* = 0.005). For girl and boy drinkers, nearly half (49.5% and 44.1%, respectively) met the criteria for problem drinkers, measured using the Cut-Annoyed-Guilty-Eye-Opener (CAGE) questionnaire. Conclusions. The high prevalence of alcohol use and problem drinking among youth, as well as alcohol-related harm, warrant urgent alcohol prevention and intervention strategies, particularly among these underserved girls.

## 1. Introduction

Alcohol is one of the most commonly used substances globally and contributes to roughly 5.3% of deaths and 5.1% of the burden of global disease [1]. While the evidence of the range of health consequences linked to alcohol is growing, numerous diseases and health conditions have already been identified [2,3]. However, of greatest importance for adolescents and young adults are the acute consequences of alcohol use that are related to unintentional injuries, including road traffic crashes, falls, and drowning, as well as unprotected sex and interpersonal and self-directed violence [2,3].

Alcohol use among youth has been linked to a range of risky behaviors; such as engaging in unprotected sex and contracting sexually transmitted infections (STIs) [4,5,6,7,8]. Alcohol use is also a known risk factor for dating and intimate partner violence (both victimization and perpetration) [9], and the link between alcohol use and intimate partner violence also presents a mechanism of HIV and STI acquisition through forced sex [10,11]. While there is a vast literature on alcohol use and alcohol-related risky behaviors among youth in high-income countries; there is a dearth of research on alcohol use among youth in low-income countries [12]. Youth living on the streets or in slums in low-income countries are particularly vulnerable to adverse health problems and risk behaviors, due to their dire living conditions. Alcohol use among these youth may exacerbate other health problems and increase involvement in health risk behaviors [13,14]. For example; among street youth in Ghana; alcohol use was relatively common; and 12% of youth reported daily alcohol use [15]. In that study; alcohol use in the previous month was also linked to four measures of sexual risk behavior: ever having sex, survival sex, sex with multiple partners, and non-condom use [15]. A recent review paper on youth in Eastern Africa reported a 52% median prevalence of alcohol use in the past month [16]. An estimated 15% (median prevalence) of youth also reported problem drinking [16]

Uganda has very high alcohol consumption rates, with an estimated 9.5 liters of alcohol consumed per person over 15 years of age [1]. This is drastically higher than the world consumption rate of alcohol (6.4 liters) [1]. Even so, epidemiologic research on alcohol use patterns in Uganda remains relatively scarce. However, Weiss and colleagues reported a 78% alcohol use prevalence among Ugandan women who were at heightened risk for HIV acquisition [17]. Among the women who reported alcohol use, 56% were classified as problem drinkers [17]. Our previous smaller scale studies have documented a high prevalence of alcohol use and drunkenness (33.9%) among youth living in the slums of Kampala, Uganda [14,18,19,20]. Our previous research has also reported an association between alcohol use and commercial sex work [21], violence [18,22], intimate partner violence [23], HIV and STIs [21,24], alcohol-related child physical abuse [25] and suicidal ideation [20,26], among youth living in the slums of Kampala.

Despite an increase in alcohol marketing reaching youth in Uganda, (marketing which is largely unregulated and includes the distribution of free alcohol and alcohol-branded merchandise) [14,21,27,28], limited research has described and examined the alcohol consumption patterns among vulnerable youth. While several studies have documented alcohol use among youth in the slums of Kampala in the context of marketing [14], commercial sex work [21,29], mental health [20,26], as well as alcohol and risky behaviors more broadly [19,22,25], there is no study, to our knowledge, documenting the overall frequency of alcohol use, problem drinking and alcohol-related harm among boys and girls living in the slums of Kampala, Uganda. This information is urgently needed to inform prevention and intervention strategies.

The purpose of this study is to add to the limited knowledge about the prevalence of alcohol use, problem drinking, and alcohol-related harm, among boys and girls living in the slums of Kampala, Uganda. The current study expands on previous studies, by presenting data from a larger study population, representing both boys and girls who are living in the slums of Kampala [14,17,18,19]. This study specifically examines the context for alcohol use, alcohol use patterns and problem drinking that may accompany alcohol harm. The current study also determines the gender differences in alcohol use, problem drinking and alcohol-related harm.

## 2. Materials and Methods

### 2.1. Setting

The Kampala Youth Survey was a cross-sectional survey conducted in March and April 2014 to examine risk behaviors and exposures, with a primary focus on alcohol use, violence, sexual risk behaviors and HIV, in a sample of youth who were, at that time, living in the slums of Kampala, 12–18 years of age. At the time of the survey, the youth were participating in a Uganda Youth Development Link (UYDEL) drop-in center. The urban UYDEL centers provide vocational training, reproductive health services, and psycho-social counseling for disadvantaged youth who are living on the streets or in the slums of Kampala [30]. Study participants comprised a non-probability sample of youth, included service-seeking youth who were recruited at six of the urban drop-in centers as well as the neighborhoods surrounding the drop-in centers. As such, our participants do not represent a random sample and the overall representativeness cannot be assured.

### 2.2. Data Collection

Over the 2-week data collection period, 1133 young people completed the entire survey. The survey was administered to the participants on Google Nexus 7 tablets using Qualtrics survey software (Qualtrics, Provo, Utah, USA). The use of tablets as an mHealth technology allowed for the easier administration of the survey and streamlined data collection. UYDEL interviewers received a one-day training on survey administration protocols and the use of the tablet. Survey questions were translated into Luganda (the most common language) by a certified translator and back-translated for accuracy, so that the survey could be offered in both English and Luganda. In-person interviews lasting 20–30 minutes were conducted by UYDEL staff after proper consenting procedures (parental consent had been waived). Participation was limited to youth ages 12–18. There were no exclusion criteria. Recruited youth received a small snack and a drink (juice or soda) as incentives for participating in the survey. Institutional Review Board approvals were obtained from Georgia State University and the Uganda National Council on Science and Technology (Approval code: SS3338).

The Kampala Youth Survey 2014 was composed of questions examining alcohol use and exposure to alcohol marketing, physical and sexual violence perpetration and victimization, sexual risk behaviors and mental health symptoms among adolescents. The questions included in the survey were collected from previously validated instruments, including: Global School-based Student Health Survey (GSHS) [31], Kampala Youth Survey 2011 [18,19,20,32], MAMPA 2012 Questionnaire, AUDIT Questionnaire [33], CAGE Questionnaire [34], iMPPACS [35], AIDS Indicator Survey [36], and the Demographic Health Survey [37].

### 2.3. Data Analysis

Descriptive statistics were computed for the frequency of alcohol use, alcohol use consumption patterns, and alcohol-related behaviors. Responses were stratified by gender and also for problem drinkers compared to non-problem drinkers. Problem drinkers and non-problem drinkers were classified using the CAGE questionnaire [34]. The CAGE questionnaire consists of four questions (“Have you ever felt you should cut down on your drinking?”; “Have people annoyed you by criticizing your drinking?”; “Have you ever felt bad or guilty about your drinking?”; and “Have you ever had a drink first thing in the morning to steady your nerves or to get rid of a hangover (eye opener)?”) [34]. Items are scored 0 or 1, and a higher score indicates a more severe alcohol problem. Scores of 2 or more are considered “clinically significant,” and participants that score this high are classified as problem drinkers [34]. Chi-square tests were used to determine differences of alcohol use behaviors between (1) gender (boys vs. girls), and (2) alcohol use behaviors between problem drinkers and non-problem drinkers, stratified by gender. Additionally, we conducted a subgroup analysis, to determine if gender differences between alcohol use behaviors varied for younger versus older youth.

Finally, logistic regression analyses were conducted to determine the odds of being classified as a problem drinker by CAGE scores (dependent variable). Independent variables include demographic variables (age and education), orphan status, homelessness, and the age at first alcohol consumption. Analyses were restricted to only drinkers (*n* = 346). These analyses were separated by gender and finally conducted together in the same model, with gender as a covariate. All analyses were performed using SAS 9.4 (SAS Institute Inc., Cary, NC, USA)

## 3. Results

Among all respondents (*n* = 1133), the prevalence of any alcohol use in the past 12 months was 31% (*n* = 346) (Table 1).

Among youth who reported any alcohol use in the past 12 months, the majority were girls (55%), with a median age of 17 years (Interquartile Range: 2), had a secondary education or higher (42%), and reported one parent dead (43%). However, it should be noted that the majority of the overall sample consisted of girls. There were no statistically significant differences in alcohol users compared to non-alcohol users, with respect to gender and education. However, a statistically significant difference was detected between alcohol users and non-alcohol users for parental living status ($\chi^{2}$ = 33.74, *df* = 2, *p* < 0.01). A higher proportion of youth reported both parents dead among youth who reported any alcohol use, compared to youth who did not report any alcohol use (29% vs. 19%, respectively). Additionally, the prevalence of youth who ever lived on the streets was twice as high for youth who reported alcohol use, compared to youth who did not report alcohol use (39% vs. 15%, respectively). This difference was also statistically significant ($\chi^{2}\;$= 81.51, *df* = 1, *p* < 0.01).

Descriptive statistics and Chi-square statistics for alcohol use behaviors among boys and girls are presented in Table 2.

No statistically significant differences were found between girls and boys with regards to alcohol use in the past year. A higher percentage of boys reported the age of first alcohol beverage consumption between the ages of 1–14 compared to girls (18% vs. 13%, respectively). The majority of youth reported that either a friend or a sexual partner provides them with alcoholic beverages. Boys and girls most frequently reported the age of first drunkenness at 15–16 years of age (37% and 36%, respectively). The most commonly consumed alcoholic beverages among girls were beer, lager, or stout beer (65%), whereas among of boys they most commonly consumed local spirits (63%). The majority of drinkers reported drinking with their friends or sexual partners (84%), and this was similar for girls (81%) and boys (87%). However, a much higher percentage of girl drinkers reported drinking alcohol with a sexual partner (18%) compared to boys (2%). There were no significant differences between boys and girls with regards to alcohol use in the past year, age at first consumption of alcoholic beverage, drinking partners, and frequency of alcoholic beverage consumption.

Age stratification results for alcohol use behaviors between girls and boys were similar for alcohol use behaviors, with a few exceptions. The frequency of alcohol beverage consumption was statistically significantly different between boys and girls among the older youth, ($\chi^{2}\;$= 9.65, *df* = 3, *p* = 0.02). While a higher percentage of boys reported drinking most frequently (4 or more times a week—14.0% compared to girls 9.9%), girls reported a higher prevalence of drinking 2–3 times compared to boys (45.4% vs. 26.2%, respectively). This was not statistically significant for the overall sample.

The consequences of alcohol consumption among youth who reported alcohol use in the past 12 months, (*n* = 346), stratified by gender, are reported in Table 3.

Among boy drinkers, a higher percentage (51%) reported getting in a fight compared to girl drinkers (41%). These differences were not statistically significant. Boy and girl drinkers were similar in responses for alcohol consequences with regards to injuries, social, or work situations, including getting into an accident, having serious problems with parents and friends, performing badly at work or school, being a victim of robbery, seriously injuring themselves or others, and having to go to a hospital due to alcohol use. However, boy and girl drinkers were statistically different in reporting the consequences of alcohol consumption with regards to sexual experiences and getting in trouble with police. A higher percentage of boy drinkers reported having trouble with the police due to alcohol use (24%) compared to girl drinkers (14.6). These gender differences were statistically significant ($\chi^{2}\;$= 4.86, *df* = 1, *p* = 0.03). Additionally, a higher percentage of girl drinkers reported having sex in the past month without a condom (58%) due to alcohol consumption, compared to boy drinkers (42%). These gender differences were also statistically significant ($\chi^{2\;}$= 8.09, *df* = 1, *p* = 0.005). A higher percentage of girl drinkers also reported having sex which they wished they had not the next day due to alcohol consumption (55%), compared to boy drinkers (40%) ($\chi^{2}\;$= 7.41, *df* = 1, *p* = 0.006).

Results from the age stratification for alcohol-related harm were similar for older youth and younger youth, with regards to injuries, social, or work situations, including getting into an accident, having serious problems with parents and friends, performing badly at work or school, being a victim of robbery, seriously injuring themselves or others, and having to go to a hospital due to alcohol use. Additionally, while the overall sample yielded statistically significant differences between boys and girls with regards to sex-related consequences, the stratified sample of older youth did not.

Table 4 presents alcohol use behaviors, comparing problem drinkers to non-problem drinkers among girls and among boys.

For girls and boys, nearly half of drinkers (50.5% and 44.1%, respectively) met the criteria for problem drinkers measured using the CAGE questionnaire [34]. While a slightly higher percentage of girl drinkers were classified as problem drinkers compared to boy drinkers, among the overall sample of boys and girls, this difference was not observed. Additionally, among all males, a higher percentage were classified as problem drinkers (30.6%), compared to among all girls (15.3%). Among girl drinkers, more problem drinkers than non-problem drinkers reported that a friend, relative, or doctor suggested that they cut down on drinking (48.5% vs. 23.2%, respectively) ($\chi^{2}$= 12.98, *df* = 1, *p* < 0.01). A similar difference between problem drinkers and non-problem drinkers was also observed in boy drinkers ($\chi^{2}$= 33.13, *df* = 1, *p* < 0.01).

Lastly, Table 5 presents logistic regression results for problem drinking among youth living in the slums of Kampala. These analyses were also stratified by gender. 

For the model examining female drinkers, being classified as a problem drinker was associated with reporting that both parents were dead in the unadjusted association (OR: 2.25; 95% CI: 1.02, 4.98); however, this was not statistically significant in the multivariable model. For the model examining male drinkers, being classified as a problem drinker was also associated with reporting that both parents were dead in the unadjusted association (OR: 2.34; 95% CI: 1.00, 5.46), which was also statistically significant in the multivariable model (OR: 4.23; 95% CI: 1.57, 11.34). No other variables were statistically significantly associated with problem drinking in the separate gender models. In the collective multivariable model, problem drinking was associated with age (OR: 1.24; 95% CI: 1.01, 1.52), reporting one parent alive (OR: 1.97; 95% CI: 1.14, 3.42), and reporting both parents dead (OR: 2.54; 95% CI: 1.36, 4.76), after adjusting for gender, homelessness, and age of first alcoholic beverage.

## 4. Discussion

In this study, we examined the frequency of alcohol use, problem drinking, and alcohol-related harm among youth living in the slums of Kampala. Nearly one-third of youth reported alcohol consumption in the past 12 months, and approximately half of these youth met the criteria for problem drinking. The prevalence of alcohol use among youth in our population was nearly 2.5 times higher than the alcohol use prevalence among street youth in Ghana [15]. Problem drinking among youth was also three times higher in our study compared to street youth in Ghana [15]. However, the prevalence of alcohol use among youth in our study was lower than the median prevalence of alcohol use (52%), documented in a recent review of youth in Eastern Africa [16]. Additionally, the prevalence of alcohol use in our study was much lower than the prevalence of alcohol use among women living in Uganda (78%), but the age-specific rates of problem drinking among girls were similar [17]. It should be noted, however, that the sample of women in the previous study was slightly older (14 years of age and older) [17], compared to the current study (12–18 years).

A relatively high percentage of girls in our study reported alcohol use and alcohol-related consequences. More specifically, a greater percentage of girls compared to boy drinkers reported consuming alcohol with a sexual partner, and a higher percentage of girls compared to boy drinkers also reported having sex which they regretted the next day and having sex without a condom due to alcohol use. While these findings were evident among the older youth, this difference was also apparent in the overall sample. This is a serious concern, particularly because of the high rates of HIV/AIDS and sexually transmitted diseases among youth living in the slums of Kampala [19,21]. 

Among the youth who reported drinking, problem drinking was associated with orphan status in both the separate gender logistic models and the overall logistic model. Alcohol prevention interventions for orphans should be examined for this population, as orphans are also at a heightened risk of experiencing other adverse health outcomes [38].

The prevalence of consequences related to alcohol use was relatively high among both girls and boys. Both sexes reported unintentional injuries to themselves and others, risky sexual behaviors, and social and work problems, due to alcohol use. Targeted interventions and alcohol prevention initiatives are urgently needed to prevent alcohol consumption among youth and to decrease the amount of alcohol-related disease and mortality burden. 

### Limitations

The limitations of our study include the convenience sample of youth. As such, our findings may not be generalizable to the broader sample of youth living in the slums of Kampala or to other youth populations in sub-Saharan Africa. However, one of our key finding demonstrates a high proportion of problem drinking among those drinking alcohol, which is of great concern for public health prevention and intervention strategies. Moreover, since all measures were self-reported, results may be influenced by self-reporting and social desirability bias. Since interviewers were familiar with the youth, social desirability bias would potentially have a substantial impact on results. Risky behaviors and alcohol use prevalence may be underreported. Future research should investigate the potential biases of self-reported alcohol use in this population, with biomarkers and other tools such as daily diaries that can validate self-reported measures. 

## 5. Conclusions

Despite our limitations, this study is the first to our knowledge to document the overall public health problem of alcohol use behaviors among boys and girls and alcohol-related consequences among youth living in the slums of Kampala, Uganda. This study presents important findings on a hard-to-reach and understudied population, that is heavily exposed to alcohol marketing, in order to inform prevention initiatives [14]. The high prevalence of alcohol use and alcohol-related consequences among these youth warrant immediate attention. 

Most importantly, a large proportion of youth in our study were under the legal drinking age for Uganda (age 18). As such, our findings underscore the need for underage drinking policies and enforcement of the legal drinking age. Awareness campaigns should target youth, particularly those out of school, and emphasize the direct and indirect harms of alcohol consumption. Future research should also investigate risk and protective factors associated with alcohol use among youth living in the slums of Kampala, to further inform prevention initiatives. Cohort and longitudinal studies are needed to further understand the trajectories of alcohol use and harm among youth and the impact of alcohol use in this population.

## Figures and Tables

**Table 1:** Demographic characteristics of youth in the 2014 Kampala Youth Survey, (*n* = 1133).

Variable Name	Alcohol Use—Yes (*n* = 346) 30.54%	Alcohol use—No ( *n* =787) 69.46%	Total	Chi-Square, (*df*), *p*-Value
**Gender**				0.18, (1), *p* = 0.68
Boys	155 (44.8%)	342 (43.5%)	497 (43.9%)	
Girls	191 (55.2%)	445 (56.5%)	636 (56.1%)	
**Age, Median (IQR)**	17 (2)	16 (3)	17 (3)	
**Education**				0.75, (2), *p* = 0.69
Primary or less	123 (35.9%)	274 (35.3%)	397 (35.5%)	
Completed primary	75 (21.9%)	188 (24.2%)	263 (23.5%)	
Secondary or higher	145 (42.3%)	315 (40.5%)	460 (41.1%)	
**Parental living status**				33.74, (2), *p* < 0.0001
Both parents dead	101 (29.2%)	150 (19.0%)	251 (22.1%)	
One parent dead	148 (42.8%)	277 (35.2%)	425 (37.5%)	
Both parents living	97 (28.0%)	361 (45.8%)	458 (40.4%)	
**Ever lived on the streets with nowhere to go**				81.51, (1), *p* < 0.0001
Yes	134 (38.7%)	115 (14.6%)	249 (22.0%)	
No	212 (61.3%)	672 (85.4%)	884 (78.0%)	

**Table 2:** Alcohol use prevalence and alcohol use behaviors among youth living in the slums of Kampala by gender, (*n* = 1133).

Variable	Boys (*n* = 497) 43.87%	Girls (*n* = 636) 56.13%	Total *n* = 1133	Chi-Square, *(df), p*-Value
**Alcohol use in the past year**				0.18, (1), *p* = 0.68
Yes	155 (31.2%)	192 (30.0%)	346 (30.5%)	
No	342 (68.8%)	445 (70.0%)	787 (69.5%)	
**Age at consumption of first alcoholic beverage**				5.93, (3), *p* = 0.11
1–14	90 (18.2%)	84 (13.3%)	174 (15.5%)	
15–16	66 (13.3%)	99 (15.7%)	165 (14.7%)	
17–18	26 (5.3%)	40 (6.3%)	66 (5.9%)	
Non-drinker	313 (63.2%)	407 (64.6%)	720 (64.0%)	
**Who usually gives you alcohol to drink?**				7.53, (2), *p* = 0.02
Family (parents, siblings, etc.)	9 (10.6%)	16 (8.3%)	25 (7.2%)	
Friends or sex partners	9 (10.6%)	120 (62.5%)	199 (57.4%)	
I get it myself or other	67 (78.8%)	56 (29.2%)	123 (35.5%)	
**Age at first drunkenness**				8.27, (3), *p* = 0.04
12–14	53 (34.2%)	50 (26.0%)	103 (29.7%)	
15–16	57 (36.8%)	69 (35.9%)	126 (36.3%)	
17–18	22 (14.2%)	50 (26.0%)	72 (20.8%)	
Never	23 (14.8%)	23 (12.0%)	46 (13.3%)	
Type of alcohol usually consumed *				
Beer, lager, or stout	67 (35.1%)	124 (64.9%)	191 (55.0%)	
Wine	10 (55.6%)	8 (44.4%)	18 (5.1%)	
Local spirits	26 (63.4%)	15 (36.6%)	41 (11.8%)	
Distilled spirits	43 (55.1%)	35 (44.9%)	78 (22.5%)	
Local brews	9 (52.9%)	8 (47.1%)	17 (4.9%)	
**Whom do you usually drink alcohol with?**				2.65, (2), *p* = 0.27
With my friends or sexual partners	135 (87.1%)	156 (81.3%)	291 (83.9%)	
With my family	8 (5.2%)	18 (9.4%)	26 (7.5%)	
With others or alone	12 (7.7%)	18 (9.4%)	30 (8.7%)	
**Frequency of alcoholic beverage consumption**				5.36, (3), *p* = 0.15
Monthly or less	35 (22.73%)	35 (18.23%)	70 (20.23%)	
2–4 times a month	49 (31.82%)	55 (28.65%)	104 (30.06%)	
2–3 times a week	47 (30.52%)	81 (42.19%)	128 (36.99%)	
4 or more times a week	23 (14.94%)	21 (10.94%)	44 (12.72%)	
**Number of drinks typically consumed during a drinking day**				1.05, (3), *p* = 0.79
1–2 drinks	89 (57.79%)	106 (55.50%)	195 (56.52%)	
3–4 drinks	49 (31.82%)	69 (36.13%)	118 (34.20%)	
5 or more drinks	16 (10.39%)	16 (8.38%)	32 (9.28%)	

Note. * Chi-square/Fisher exact tests were not performed on this category, because the categories are not mutually exclusive and were measured using different items.

**Table 3:** Alcohol use behaviors among youth living in the slums of Kampala by gender among youth ages 16–18, (*n* = 600).

Variable	Boys (*n* = 269) 44.8%	Girls (*n* = 331) 55.2%	Total	Chi-Square, *(df), p*-Value
			*n* = 600	
**Alcohol use in the past year**				0.28, (1), *p* = 0.60
Yes	108 (40.1%)	140 (42.3%)	248 (41.3%)	
No	161 (59.9%)	191 (57.7%)	352 (58.7%)	
**Age at consumption of first alcoholic beverage**				2.97, (3), *p* = 0.40
1–14	45 (16.9%)	42 (12.7%)	87 (14.6%)	
15–16	56 (21.0%)	78 (23.6%)	134 (22.5%)	
17–18	26 (9.7%)	40 (12.1%)	66 (11.1%)	
Non-drinker	140 (52.4%)	170 (51.5%)	310 (51.9%)	
**Who usually gives you alcohol to drink?**				0.04, (1), *p* = 0.84
Family or friends	53 (49.1%)	71 (50.4%)	124 (49.8%)	
Sex partners, myself or others	55 (50.9%)	70 (49.7%)	125 (50.2%)	
**Age at first drunkenness**				8.91, (3), *p* = 0.03
12–14	24 (22.2%)	25 (17.7%)	49 (19.7%)	
15–16	44 (40.7%)	54 (38.3%)	98 (39.4%)	
17–18	22 (20.4%)	50 (35.5%)	72 (28.9%)	
Never	18 (16.7%)	12 (8.5%)	30 (12.1%)	
Type of alcohol usually consumed *				
Beer, lager, or stout	49 (34.3%)	93 (65.7%)	143 (57.9%)	
Wine	7 (46.7%)	8 (53.3%)	15 (6.1%)	
Local spirits	13 (59.1%)	9 (40.9%)	22 (8.9%)	
Distilled spirits	33 (58.9%)	23 (41.1%)	56 (22.7%)	
Local brews	6 (54.6%)	5 (45.6%)	11 (4.5%)	
**Whom do you usually drink alcohol with?**				0.28, (1), *p* = 0.60
With my family, friends, or sex partners	100 (37.2%)	130 (39.3%)	230 (38.3%)	
With others or alone	169 (62.8%)	201 (60.7%)	370 (51.7%)	
**Frequency of alcoholic beverage consumption**				9.65, (3), *p* = 0.02
Monthly or less	25 (23.4%)	25 (17.7%)	50 (20.2%)	
2–4 times a month	39 (36.5%)	38 (27.0%)	77 (31.1%)	
2–3 times a week	28 (26.2%)	64 (45.4%)	92 (37.1%)	
4 or more times a week	15 (14.0%)	14 (9.9%)	29 (11.7%)	
**Number of drinks typically consumed during a drinking day**				1.74, (2), *p* = 0.42
1–2 drinks	61 (24.7%)	69 (22.3%)	130 (23.3%)	
3–4 drinks	35 (14.2%)	57 (18.4%)	92 (16.5%)	
5 or more drinks	11 (4.5%)	14 (4.5%)	25 (4.5%)	
**Has a relative, friend, doctor, or other healthcare worker been concerned about your drinking or suggested you cut down?**				2.67, (1), *p* = 0.10
Yes	35 (32.4%)	60 (42.6%)	95 (38.2%)	
No	73 (67.6%)	81 (57.5%)	154 (61.9%)	
**Days of alcohol consumption in the past month**				0.91, (3), *p* = 0.82
0 days	7 (6.6%)	6 (4.3%)	13 (5.3%)	
1 or 2 days	41 (38.7%)	53 (37.6%)	94 (38.1%)	
3 to 5 days	30 (28.3%)	45 (31.9%)	75 (30.4%)	
6+ days	28 (26.4%)	37 (26.2%)	65 (26.3%)	
**Days of having a hangover, feeling sick, getting into trouble with friends or family, miss school, or get into fights because of drinking alcohol?**				4.72, (3), *p* = 0.19
0 days	36 (33.6%)	34 (24.1%)	70 (28.2%)	
1 or 2 days	43 (40.2%)	71 (50.4%)	114 (46.0%)	
3 to 5 days	21 (19.6%)	22 (15.6%)	43 (17.3%)	
6+ days	7 (6.5%)	14 (9.9%)	21 (8.5%)	
**How many times (if any) have you had five or more drinks on one occasion?**				3.85, (3), *p* = 0.28
0 days	30 (28.3%)	44 (31.2%)	74 (30.0%)	
1 or 2 days	33 (31.1%)	52 (36.9%)	85 (34.4%)	
3 to 5 days	29 (27.4%)	36 (25.5%)	65 (26.3%)	
6+ days	14 (13.2%)	9 (6.4%)	23 (9.3%)	

Note.* Chi-square/Fisher exact tests were not performed on this category, because the categories are not mutually exclusive and were measured using different items. Small cell sizes for this category “Other” were suppressed.

**Table 4:** Reported consequences of alcohol consumption among youth living in the slums of Kampala who reported alcohol use in the past 12 months, (*n* = 346).

Variable	Boys	Girls	Total	Chi-Square, (df),
	*n* = 155	*n* = 192	*n* = 346	*p*-Value
	(31.20%)	(30.00%)		
**Because of your own alcohol use, how often during the** **last month have you experienced any of the following?**				
**Got in a fight**				3.34, (1), *p* = 0.07
No	76 (49.0%)	113 (58.9%)	189 (54.5%)	
Yes	79 (51.0%)	79 (41.1%)	158 (45.5%)	
**Got in an accident**				0.002, (1), *p* = 0.96
No	119 (76.8%)	147 (76.6%)	266 (76.7%)	
Yes	36 (23.2%)	45 (23.4%)	81 (23.3%)	
**Had serious problems with your parents**				0.12, (1), *p* = 0.73
No	103 (66.5%)	131 (68.2%)	234 (67.4%)	
Yes	52 (33.5%)	61 (31.8%)	113 (32.6%)	
**Had serious problems with your friends**				3.92, (1), *p* = 0.05
No	69 (44.5%)	106 (55.2%)	175 (50.4%)	
Yes	86 (55.5%)	86 (44.8%)	172 (49.6%)	
**Performed badly at work or school**				1.31, (1), *p* = 0.25
No	106 (68.4%)	142 (74.0%)	248 (71.5%)	
Yes	49 (31.6%)	50 (26.0%)	99 (28.5%)	
**Was a victim by robbery or theft**				0.02, (1), *p* = 0.88
No	116 (74.8%)	145 (75.5%)	261 (75.2%)	
Yes	39 (25.2%)	47 (24.5%)	86 (24.8%)	
**Had trouble with police**				4.86, (1), *p* = 0.03
No	118 (76.1%)	164 (85.4%)	282 (81.3%)	
Yes	37 (23.9%)	28 (14.6%)	65 (18.7%)	
**Had to go to a hospital**				1.57, (1), *p* = 0.21
No	133 (85.8%)	155 (80.7%)	288 (83.0%)	
Yes	22 (14.2%)	37 (19.3%)	59 (17.0%)	
**Had sex without a condom**				8.09, (1), *p* = 0.005
No	90 (58.1%)	82 (42.7%)	172 (49.6%)	
Yes	65 (41.9%)	110 (57.3%)	175 (50.4%)	
**Had sex which you wished you hadn’t the next day**				7.41, (1), *p* = 0.006
No	93 (60.0%)	87 (45.3%)	180 (51.9%)	
Yes	62 (40.0%)	105 (54.7%)	167 (48.1%)	
**Had sex with multiple partners**				0.61, (1), *p* = 0.44
No	106 (68.4%)	123 (64.4%)	229 (66.2%)	
Yes	49 (31.6%)	68 (35.6%)	117 (33.8%)	
**Ever been seriously injured or hurt due to your drinking?**				0.70, (1), *p* = 0.40
Yes	55 (35.7%)	77 (40.1%)	132 (38.2%)	
No	99 (64.3%)	115 (59.9%)	214 (61.8%)	
**Has someone else ever been seriously injured or hurt due to your drinking?**				0.005, (1), *p* = 0.95
Yes	42 (27.3%)	53 (27.6%)	95 (27.5%)	
No	112 (72.7%)	139 (72.4%)	251 (72.5%)	
**Ever sought help for your drinking?**				1.10, (1), *p* = 0.29
Yes	30 (19.4%)	29 (15.1%)	59 (17.0%)	
No	125 (80.6%)	163 (84.9%)	288 (83.0%)	

**Table 5:** Logistic regression results for problem drinking among youth living in the slums of Kampala, Uganda, stratified by gender, (*n* = 346).

Variable	Girls		Boys		All	
	(*n* = 191)		(*n* = 155)		(*n* = 346)	
	**Unadjusted OR**	**Adjusted OR**	**Unadjusted OR**	**Adjusted OR**	**Unadjusted OR**	**Adjusted OR**
**Gender**						
Male					1	1
Female					1.31 (0.85, 2.01)	1.22 (0.78, 1.92)
**Age**	1.22 (0.95, 1.56)	1.34 (0.99, 1.83)	1.24 (0.97, 1.59)	1.12 (0.83, 1.53)	1.24 (1.04, 1.47)	1.24 (1.01, 1.52)
**Education**						
Less than primary	1	1	1	1	1	1
Completed primary	0.38 (0.17, 0.87)	0.40 (0.17, 0.95)	1.03 (0.44, 2.44)	1.29 (0.49, 3.44)	0.60 (0.33, 1.08)	0.65 (0.35, 1.20)
Secondary or higher	0.95 (0.50, 1.82)	1.00 (0.50, 2.01)	1.73 (0.82, 3.64)	1.89 (0.75, 4.78)	1.25 (0.77, 2.02)	1.31 (0.76, 2.25)
**Homelessness**						
Yes	1.57 (0.87, 2.85)	1.36 (0.70, 2.63)	0.92 (0.48, 1.76)	0.90 (0.43, 1.90)	1.21 (0.78, 1.87)	1.14 (0.71, 1.85)
No	1	1	1	1	1	1
**Orphan Status**						
Both parents alive	1	1	1	1	1	1
One parent alive	2.00 (0.96, 4.17)	1.97 (0.92, 4.21)	1.99 (0.91, 4.34)	2.01 (0.87, 4.64)	2.04 (1.20, 3.48)	1.97 (1.14, 3.42)
Both parents dead	2.25 (1.02, 4.98)	1.98 (0.84, 4.64)	2.34 (1.00, 5.46)	4.23 (1.57, 11.34)	2.33 (1.31, 4.15)	2.54 (1.36, 4.76)
**Age of first alcohol beverage**						
1–14	1.65 (0.72, 3.79)	2.68 (0.97, 7.41)	0.68 (0.25, 1.83)	0.69 (0.22, 2.13)	1.07 (0.57, 2.00)	1.42 (0.69, 2.96)
15–16	1.57 (0.70, 3.49)	1.68 (0.72, 3.93)	1.83 (0.67, 5.04)	1.89 (0.64, 5.58)	1.67 (0.90, 3.12)	1.72 (0.89, 3.32)
17–18	1	1	1	1	1	1

Note. Statistically significant associations are bolded.
